# C,N-codoped MoSi_2_ ceramic with excellent heat resistance for microwaves absorption application

**DOI:** 10.1098/rsos.200740

**Published:** 2020-07-29

**Authors:** Zhiqian Yang, Chang Xu, Yilu Xia, Ziming Xiong

**Affiliations:** State Key Laboratory for Disaster Prevention and Mitigation of Explosion and Impact, Army Engineering University of PLA, Nanjing 210007, People's Republic of China

**Keywords:** MoSi_2_ (high-temperature ceramic), multi-polarization resonance, microwaves absorption

## Abstract

Microwave absorption (MA) materials with high heat resistance have a wide range of applications in many fields. In this work, a C,N-codoped MoSi_2_ ceramic was prepared via a facile solid-phase reaction method and its MA properties was investigated. On the one hand, the results indicate that this ceramic possesses excellent heat resistance and the weight of the MoSi_2_ is almost constant when the temperature is lower than 800°C. On the other hand, this ceramic shows good MA performance when the filler loading ratio increases to 30 vol%, the value of reflection loss (RL) could reach to −17.70 dB at 7.44 GHz with the thickness of 2.0 mm and the effective electromagnetic absorption bandwidth (RL below −10 dB) could reach to 1.88 GHz (9.28–11.16 GHz) with the thickness of 1.5 mm. Multi-polarization resonance loss is considered as the predominant attention mechanism on the MA performance of this MoSi_2_ ceramic. This research provides a new idea for understanding resonance mechanism and greatly expands the application scope of MoSi_2_ ceramic in MA area.

## 1. Introduction

Nowadays, the overdevelopment of electronics industry leads to serious electromagnetic radiation, which is mainly caused by the interference effects induced by electric and magnetic fields emanating from wide range of electrical circuitry [1,2]. Electromagnetic radiation is not only harmful to human health, but also to some sophisticated instruments. Hence, the research on microwave absorption (MA) materials gradually attracts people's attention. At present, many kinds of materials are confirmed to possess great MA performance, such as carbon materials [3–5], conductive polymers [6,7] and chiral materials [8]. However, on the one hand, temperature is one of the most important factors affecting dielectric properties [9–11]; these materials' MA performance varies greatly at different temperatures. On the other hand, these materials are not stable at exceedingly high temperature and thus restricted to extremely harsh conditions in certain applications.

In recent years, due to high temperature resistance, ultrahigh-temperature ceramic materials have gained great attention. Zhang *et al*. found that ZrB_2_ ceramic can resist high temperature and have proper reflection loss to electromagnetic wave [12]. Dou *et al*. studied that dielectric properties of N-doped SiC and explained the mechanism of polarization relaxation [13]. Besides, the research of SiC ceramic [14–16] and composite ceramics [17–20] in the field of microwave absorption has been more in-depth. Moreover, ultrahigh-temperature ceramic not only has great mechanical properties and corrosion resistance, but also has great MA properties. Owing to great comprehensive performance, ultrahigh-temperature ceramic has great potential in military and aerospace applications. As an ultrahigh-temperature ceramic, MoSi_2_ possesses high thermal conductivity (25 W m^−1^ K), moderate density (6.24 g cm^−3^), high melting point (2300 K), relatively lower coefficient of thermal expansion (8.6 × 10^−6^ K^−1^) and elevated oxidation resistance (about 1600°C) [21]. Besides, MoSi_2_ has amazing electrical conductivity, which suggests the potential in MA materials. To the best of our knowledge, however, the MA properties of MoSi_2_ remain to be explored.

In this study, we report the MA properties of a C,N-codoped MoSi_2_ ceramic, which was prepared from a solid-state reaction method [22]. The MA performance and dielectric properties of MoSi_2_ in the frequency range of 2–18 GHz were investigated in detail. The results indicate that C,N-codoped MoSi_2_ ceramic shows excellent heat resistance as well as good MA performance. A multi-polarization resonance loss model was used to investigated the attention mechanism of C,N-codoped MoSi_2_ ceramic toward MA.

## 2. Material and methods

### 2.1. Material

The anhydrous ethanol was purchased from Shanghai GENERAL-REAGENT Titan Scientific Co., Ltd, China. The MoO_2_ was purchased from Shanghai Yunfu Nanotechnology Co., Ltd, China. The Si was purchased from Beijing Hongyu New Material Technology Co., Ltd, China.

### 2.2. Synthesis of C,N-codoped MoSi_2_ ceramic

MoSi_2_ was constructed according to the following steps. MoO_2_ (36.0 g) and Si (24.0 g) were added to the jar mill in turn. Next, ethanol (2.0 ml) was added to the jar mill. Then, the above mixture was ground for 8 h. Next, the mixture was heated for 8 h at temperature of 80°C. After drying, the mixture was passed through a 60 mesh screen and put into a graphite crucible. Then, the graphite crucible was put into pressure-free sintering furnace that is full of nitrogen, the material was sintered at heating rate of 10°C min^−1^ from room temperature to 1400°C and kept at 1400°C for 1 h.

### 2.3. Characterization

The micro morphology of MoSi_2_ samples were characterized by scanning electron microscope (SEM, Hitachi, S4800) and transmission electron microscope (TEM; FEI, Tecnai G2 F20). The crystal feature of MoSi_2_ samples were investigated by instrument (XRD; Philips, X' Pert Pro) by Cu K*α* (*λ* = 1.54 Å) radiation source (30.0 mA, 40.0 kV). The result of thermostability analysis was evaluated by thermo gravimetric analyser (TGA; TA, SDT Q600) in the air. The result of X-ray photoelectron spectroscopy (XPS) was got from an apparatus (ESCALABTM 250Xi, Thermo Fisher Scientific). MoSi_2_/wax was prepared by uniformly mixing MoSi_2_ in wax and pressed into a toroidal-shaped specimen (Фin: 3.0 mm, Фout: 7.03 mm). The electromagnetic properties of MoSi_2_ were recorded using a network analyser (VNA; N5242A PNA-X, Agilent).

## 3. Results and discussion

### 3.1. Morphology and microstructure

**Figure 1.**
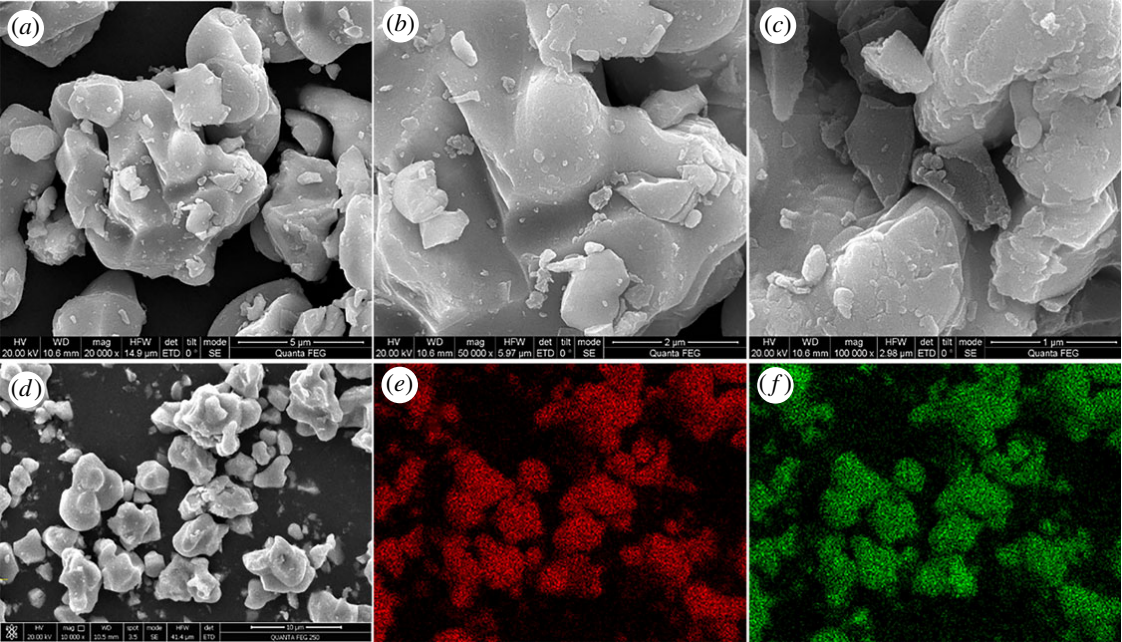
(*a*–*d*) Representative SEM image of MoSi_2_ ceramic; (*e*,*f*) representative elemental mapping analysis of MoSi_2_ ceramic.

Figure 1 shows a typical morphology of MoSi_2_. Figure 1*a*–*d* displays SEM images of MoSi_2_ at different magnifications, and a lot of irregularly shaped block structure can be observed. The block size ranges from 0.5 to 8 µm. The elemental mappings of MoSi_2_ are shown in figure 1*e*,*f*. Generally, the atomic ratio of Mo/Si was about 1 : 2. However, it is suggested that the signal of Si was a little stronger than that of Mo from figure 1*e*,*f*, which suggests the ratio of Mo/Si is lower than 1 : 2. Besides, it could be found that both Mo and Si were uniformly distributed.

**Figure 2.**
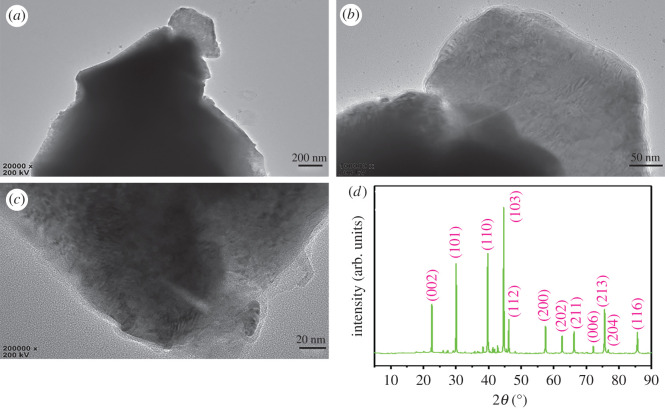
(*a*–*c*) Representative TEM image of MoSi_2_ ceramic; (*d*) XRD patterns of MoSi_2_ ceramic.

Figure 2*a*–*c* shows the inner structure of MoSi_2_ ceramics, which displays the irregularly shaped block structure was composed of layered structure by means of stacking. The XRD pattern of MoSi_2_ ceramic is exhibited in figure 2*d*. The diffraction peaks at 22.6, 30.1, 39.7, 44.6, 46.2, 57.4, 62.5, 66.2, 72.1, 75.5, 76.7 and 85.6° matched well to the (002), (101), (110), (103), (112), (200), (202), (211), (006), (213), (204) and (116) planes of MoSi_2_ (PDF#41–0612), which suggest MoSi_2_ is polycrystalline.

### 3.2. Elemental composition and thermal stability of MoSi_2_

**Figure 3.**
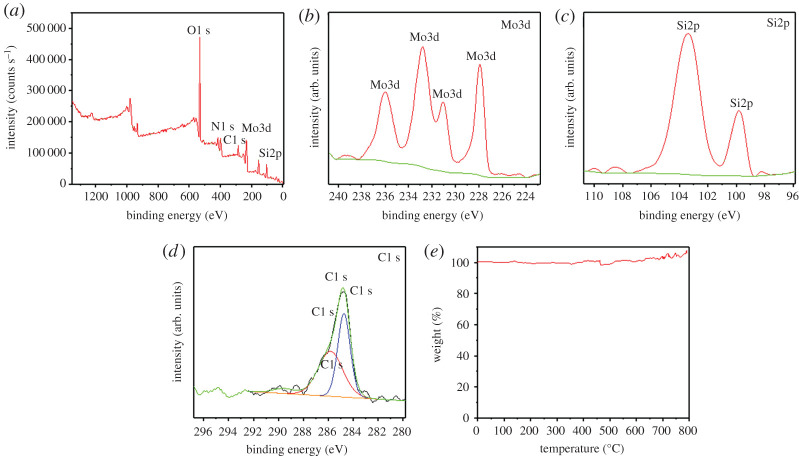
(*a*–*d*) XPS spectra of MoSi_2_ ceramic, (*e*) TGA of MoSi_2_ ceramic.

In order to further investigate the element composition and structure of MoSi_2_ ceramic, XPS was performed and is shown in figure 3*a*–*d* The peaks of C, N, O, Mo and Si can be observed in the full spectrum in figure 3*a*, the existence of O mainly due to formation of small amounts of SiO_2_. Figure 3*b*,*c* are XPS spectra of Mo and Si. Mo3d peaks are located in the range of binding energies of 236 to 227 eV and Si2p peaks are located in the range of binding energies of 104 to 99 eV. The peak of C1 s is located at binding energies of 284 eV according to figure 3*d*. The atomic ratio of Mo and Si is 21.06 : 78.94, which is close to 1 : 4. The ratio is higher than 1 : 2. It proves that the sample consists of a small amount of SiO_2_ and excessive Si. Figure 3*e* shows the thermal stability of MoSi_2_ ceramic; the weight of MoSi_2_ is almost constant when the temperature is below 800°C, which suggests that MoSi_2_ is an ultrahigh-temperaturee ceramic material.

### 3.3. Dielectric and microwave absorption properties

**Figure 4.**
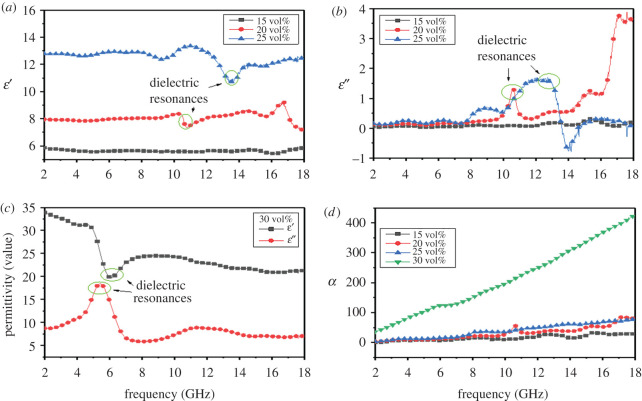
(*a*–*c*) Frequency and filler loading ratio dependence of complex permittivity; (*d*) attenuation constant (*α*) of MoSi_2_ ceramic with different filler loading ratios.

Figure 4*a*–*c* shows the real part (*ε′*) and the imaginary part (*ε″*) of the relative permittivity (*ε_r_*) of MoSi_2_ ceramic with different filler loading ratios. Generally speaking, the electromagnetic storage ability is subject to the real part (*ε′*) of complex permittivity while the energy damping is determined by the imaginary part (*ε″*) of complex permittivity [23]. As the filler loading ratio increasing, both *ε′* and *ε″* increase. When the filler loading ratio is 30 vol%, dielectric parameter of MoSi_2_ increases a lot. According to the Debye theory, the frequency is negatively correlated with the permittivity of a composite [24]. When the frequency is at 12.72, 10.56 and 5.52 GHz, resonance peaks can be observed from figure 4*a*,*b* and the value of *ε′* at peak point is greatly improved. Hence, the loss mechanism of MoSi_2_ includes polarization loss and resonance loss. Around the 12.72 GHz of resonance peaks, the *ε″* of 25 vol% addition of MoSi_2_ drops to a negative value, which suggests this material has potential to be a metamaterial. Besides, resonance peaks shift to lower frequency and become higher when the filler loading ratio increases. Figure 4*c* shows that the resonance signal is much stronger when the addition amount is 30 vol%. As shown in figure 4*d*, attenuation constant (*α*) of MoSi_2_ ceramic is enhanced with the increase of the filler loading ratio, especially the filler loading ratio increases from 20 to 30 vol%, which indicates the energy attenuation performance of MoSi_2_ is improved as the filler loading ratio increase.

The reflection loss (RL) can directly reflect the microwave-absorbing capacity of materials, which is given as [25]

3.1 $$R_{L}=20\log\frac{\left|Z_{in}-1\right|}{\left|Z_{in}+1\right|}.$$

Here, the normalized input impedance *Z_in_* of microwave absorption layer is

3.2 $$Z_{in}=Z_{0}\sqrt{\frac{\mu_{r}}{\varepsilon_{r}}}tanh\left[j\frac{2\pi }{c}\sqrt{\mu_{r}\varepsilon_{r}}fd\right],$$

where *Z*_0_ is free space impedance, complex permittivity (*ε_r_*) can be expressed as $\varepsilon_{r}=\varepsilon^{\prime }-\varepsilon^{\prime \prime }$, complex permeability (*µ_r_*) can be expressed as $\mu_{r}=1$***_,_*** because the samples are non-magnetic. *f* is frequency, *d* is sample thickness and *c* is light speed.

**Figure 5.**
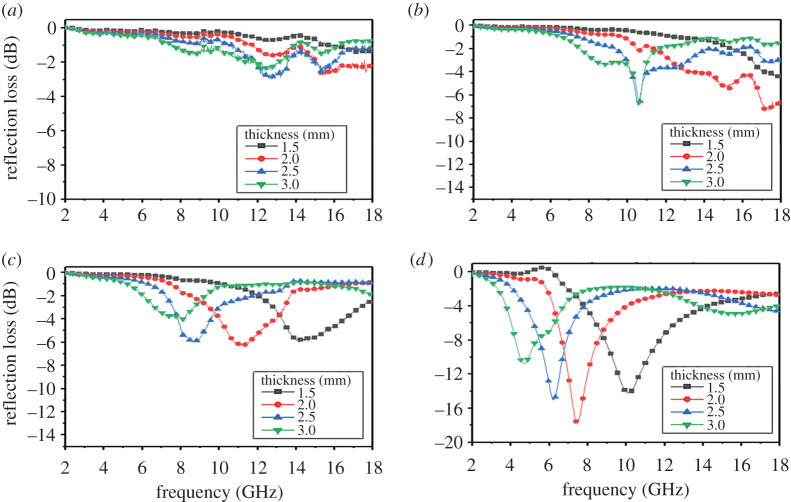
RL curves of MoSi_2_ ceramic with filler loading ratios of (*a*) 15 vol%, (*b*) 20 vol%, (*c*) 25 vol% and (*d*) 30 vol%.

Figure 5 shows the RL curves of MoSi_2_ ceramic coupled with different filler loadings and various thicknesses at the frequency range of 2–18 GHz. The absorption peaks are mainly around 12.72, 10.56 and 5.52 GHz, which attributes to the polarization resonance loss (figure 5*a*–*d*). As the filler loading ratio increases, the MA performance of MoSi_2_ is enhanced. Meanwhile, the absorption peaks shift to lower frequency, which corresponds to permittivity. This further explains that the enhancement of MA performance is attributed to polarization resonance loss. When the filler loading ratio is lower than 25 vol%, the MA performance of MoSi_2_ ceramic is low. This is owing to the fact that the amount of MoSi_2_ is not enough to form a conductive network, which leads to a low dielectric loss. However, when the filler loading ratio increases to 30 vol%, the value of RL could reach to −17.70 dB at 7.44 GHz with the thickness of 2.0 mm and the effective electromagnetic absorption bandwidth (RL below −10 dB) can reach to 1.88 GHz (9.28–11.16 GHz) with the thickness of 1.5 mm. The MA performance of MoSi_2_ is enhanced 250% compared to the MoSi_2_ with addition lower than 25 vol%. Besides, the different thickness corresponds to different absorption peaks. When the thickness becomes thinner, the absorption peaks may be shift to the low frequency. Generally, the reason for the fact that MA performance of MoSi_2_ is enhanced is the multi-polarization resonance phenomenon. This phenomenon has been proven in permittivity and reflection loss.

### 3.4. Multi-polarization resonance

**Figure 6.**
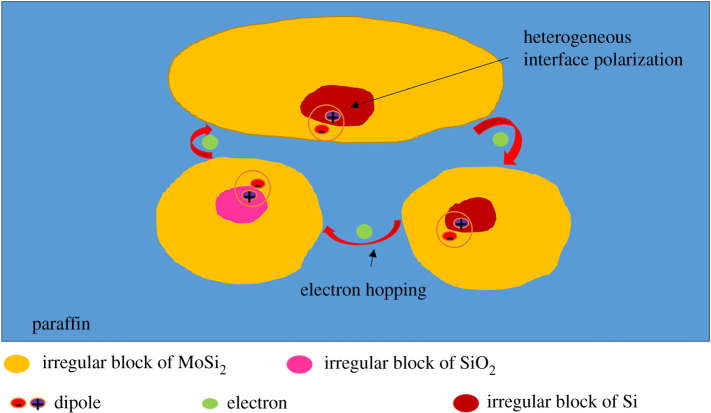
Interface effect and conductive network between SiO_2_, Si and MoSi_2_.

In general, the MA performance is related to multi-polarization resonance loss, which explains the enhanced MA performance of MoSi_2_. Figure 6 shows interface effect and conductive network between SiO_2_, Si and MoSi_2_. The heterogeneous interfacial polarization in SiO_2_, Si and MoSi_2_ is regarded as the capacitor-like structure, which is effective in the MA [26]. Besides, the electron could leap on the irregular block of MoSi_2_ after absorbing energy, which leads to the formation of conductive network of each block [26]. The existence of conductive network greatly improves the conductivity of MoSi_2_, which is attributed to the increase of the filler loading ratio. The existence of heterogeneous interface may lead to resonance loss. Resonance loss results in the MA performance of MoSi_2_ greatly improved at a certain frequency.

## 4. Conclusion

In summary, C,N-codoped MoSi_2_ ceramic was prepared by solid-state reaction method. The morphology, dielectric properties and MA performance of MoSi_2_ was investigated. The result of SEM and TEM indicate that the morphology of MoSi_2_ ceramic is irregular block structure. XPS reveals that C, N and O exist in MoSi_2_ ceramic. The curve of TGA suggests that MoSi_2_ has great thermal stability. Moreover, the result suggests that multi-polarization resonance leads to the enhanced MA performance of MoSi_2_ ceramic. As the filler loading increases, resonance signal is enhanced and resonance peak shifts to the lower frequency. Besides, the MA performance of MoSi_2_ is greatly improved at the resonance peak. When the filler loading ratio is 30 vol%, the value of RL could reach to −17.70 dB at 7.44 GHz with the thickness of 2.0 mm and the effective electromagnetic absorption bandwidth (RL below −10 dB) can reach to 1.88 GHz (9.28–11.16 GHz) with the thickness of 1.5 mm, which indicates multi-polarization resonance mechanism contributes to improvement of the MA performance of ceramic. The mechanism of multi-polarization resonance could be explained by the existence of heterogeneous interfaces in the material structure, which has been proven in XPS. This study reveals the MA performance of MoSi_2_ ceramic and suggests ultrahigh-temperature ceramic has potential for MA field.

## Supplementary Material

TGA

Reviewer comments

## Supplementary Material

XRD

## Supplementary Material

XPS

## Supplementary Material

Electromagnetic parameters

## Permission to carry out fieldwork

No permissions were required prior to conducting our research.

## Data accessibility

The raw data of TGA, XPS, XRD and electromagnetic parameters supporting this article are available at the Dryad Digital Repository: https://doi.org/10.5061/dryad.vdncjsxr7 [27].
